# A Robotic Biopsy Endoscope with Magnetic 5-DOF Locomotion and a Retractable Biopsy Punch

**DOI:** 10.3390/mi11010098

**Published:** 2020-01-17

**Authors:** Manh Cuong Hoang, Viet Ha Le, Kim Tien Nguyen, Van Du Nguyen, Jayoung Kim, Eunpyo Choi, Seungmin Bang, Byungjeon Kang, Jong-Oh Park, Chang-Sei Kim

**Affiliations:** 1 Department of Mechanical Engineering, Chonnam National University, Gwangju 61186, Korea; hmcuong.hust@gmail.com (M.C.H.); leviethavn@gmail.com (V.H.L.); nguyenkimtien90@gmail.com (K.T.N.); eunpyochoi@jnu.ac.kr (E.C.); 2 Korea Institute of Medical Microrobotics, Gwangju 61186, Koreajaya@kimiro.re.kr (J.K.); 3 Division of Gastroenterology, Department of Internal Medicine, Yonsei University College of Medicine, Seoul 120-752, Korea; bang7028@yuhs.ac

**Keywords:** active locomotive capsule endoscope, intestinal diagnosis, biopsy punching needle, biopsy capsule endoscope, electromagnetic actuation

## Abstract

Capsule endoscopes (CEs) have emerged as an advanced diagnostic technology for gastrointestinal diseases in recent decades. However, with regard to robotic motions, they require active movability and multi-functionalities for extensive, untethered, and precise clinical utilization. Herein, we present a novel wireless biopsy CE employing active five degree-of-freedom locomotion and a biopsy needle punching mechanism for the histological analysis of the intestinal tract. A medical biopsy punch is attached to a screw mechanism, which can be magnetically actuated to extrude and retract the biopsy tool, for tissue extraction. The external magnetic field from an electromagnetic actuation (EMA) system is utilized to actuate the screw mechanism and harvest biopsy tissue; therefore, the proposed system consumes no onboard energy of the CE. This design enables observation of the biopsy process through the capsule’s camera. A prototype with a diameter of 12 mm and length of 30 mm was fabricated with a medical biopsy punch having a diameter of 1.5 mm. Its performance was verified through numerical analysis, as well as in-vitro and ex-vivo experiments on porcine intestine. The CE could be moved to target lesions and obtain sufficient tissue samples for histological examination. The proposed biopsy CE mechanism utilizing punch biopsy and its wireless extraction–retraction technique can advance untethered intestinal endoscopic capsule technology at clinical sites.

## 1. Introduction

As an emerging alternative to traditional endoscopy, M2A, the first commercial capsule endoscope (CE) was approved by the U.S. Food and Drug Administration in 2001 [1]. Gradually, the CE has become a popular and reliable alternative to traditional endoscopy [2]. Miniaturized image sensors, a microprocessor, and circuit technology with low power consumption were integrated into the CE [3,4], which allowed the development of ingestible wireless capsules for the visualization of the small-intestine mucosa [5]. Endoscopic capsules have gained momentum in the medical-device market because they are more effective than traditional techniques for the diagnosis of small-intestine disorders [2,6]. After M2A was launched by Given Imaging Ltd. (Yoqneam, Israel), more than two million capsules have been used for patients at clinical sites, for medical treatment processes [7].

However, commercialized endoscopic capsules have several limitations, including passive locomotion due to peristalsis motions, the inability to perform targeted diagnosis, the long diagnosis time, and the single visualization modality only [6]. To overcome these limitations of conventional endoscopic capsules, the magnetic actuation approach has been employed to achieve active locomotion, resulting in an active CE (ACE) [8,9]. Additionally, magnetic force and torque have been extensively researched for microrobot actuation [10,11]. When the external magnetic field acting on the permanent magnet inside the capsule body is controlled, the ACE can perform flexible movements with five degrees of freedom (DOFs). By virtue of the active locomotion ability, the development of the multi-functionality of the ACE has been accelerated for both diagnosis and therapeutics involving digestive organs.

Several advanced functions are needed for CEs, e.g., biopsy, drug delivery, and tattooing [12,13,14,15]. Among them, biopsy is a promising procedure required for the final diagnosis of suspicious intestinal regions. Several studies have been conducted to develop biopsy procedures using the micro-mechanism inside the CE. Kong et al. reported a rotational cutting razor biopsy tool that was triggered by paraffin block melting and a torsional spring [16]. Shape-memory alloys have been widely used for triggering and actuating biopsy tools [17,18]. Chen et al. introduced capsule endoscopy with a micro-clamping tool manipulated by a micromotor [19]. However, in these studies, the researchers focused on the biopsy motion and did not demonstrate an integrated capsule with a targeting ability based on active locomotion and tool visualization using a camera. Moreover, because the shape-memory alloy or the micromotor mechanism consumed excessive power, which is limited inside the CE body, providing sufficient power is another challenge to be overcome. Recently, Simi et al. reported a magnetic torsion spring mechanism for the magnetic CE [20], where the biopsy module can obtain sufficient biopsy tissue for histological analysis and not consume energy from the internal capsule battery. However, the biopsy module was too large (diameter of 9 mm and length of 24 mm) to be integrated with a real endoscopic capsule. Yim et al. used a thermal-response shape-memory material to actuate micro-tools [21,22]; however, the retrieval rate was <3% of the total released biopsy micro-tools, and external locomotion could not be achieved. Other types of biopsy tool mechanisms incorporating microrobot systems have been employed for endoscopic capsules [23,24]. However, none of them provides a complete solution for the CE, including active locomotion, visual inspection, a battery-free biopsy mechanism, and a miniaturized size. Thus, these barriers must be addressed to develop a biopsy ACE for practical clinical applications.

Herein, we propose a novel biopsy methodology utilizing a clinically available biopsy needle that can be integrated into a conventional CE. A battery-free screw mechanism is developed to actuate the biopsy punch using the external magnetic field. The punch needle is stowed inside the capsule body during locomotion to avoid undesired tissue extraction and prevent perforation. It is actively extruded to obtain biopsy samples owing to the screw mechanism that converts the rotation motion of a permanent magnet into linear motion under the rotation magnetic field. After tissue is extracted, the punch needle is retracted into the CE body, and the investigation process is continued. A distinct advantage of our design is that the biopsy procedure can be viewed through the CE’s camera. The new design of the biopsy CE enables independent magnetic actuation for both flexible locomotion and tissue acquisition. This provides a complete diagnosis procedure for the intestines, including the moving, scanning, and sampling of tissue.

The remainder of this paper is organized as follows: Section 2 presents the design of the proposed biopsy CE and that of the biopsy module. The magnetic manipulation of the locomotion and the biopsy are explained in this section. Section 3 describes the evaluation tests of the proposed biopsy capsule and the ex-vivo experimental results. A discussion is presented in Section 4, followed by the conclusions in Section 5.

## 2. Materials and Methods

### 2.1. Overall System Description

**Figure 1:**
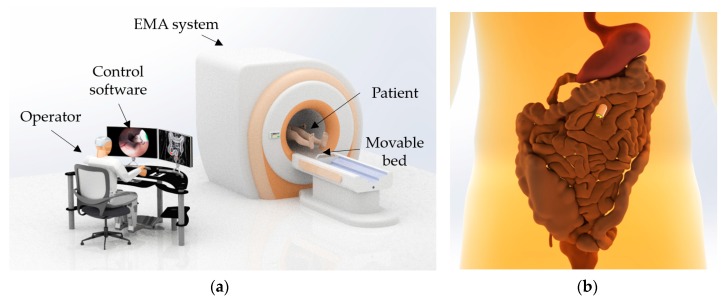
(**a**) Overview of the proposed biopsy capsule endoscope (CE) system. (**b**) Capsule movement in small intestine.

The developed biopsy CE system has two main functions: visual inspection for intestinal organ diagnosis and tissue extraction for further histological analysis. Figure 1 depicts an overview of the proposed biopsy CE system. Patients, after swallowing the function CE, lie down in a moveable bed and are then moved to the center of the electromagnetic actuation (EMA) system. The ACE is tele-operated inside the human body by the user via a joystick and the developed software. The EMA system comprises a set of electromagnets and acts as a magnetic manipulator to control the motions of the ACE. It can generate the desired magnetic field inside a region of interest (ROI) to control the movement of the capsule along planned paths. The magnetic torque created by the magnetic field is used to steer the capsule’s posture, which provides the operator multiple views for visual inspection. Meanwhile, the magnetic force resulting from the gradient magnetic field propels the capsule robot to the desired positions. Additionally, the magnetic responses of the ACE have potential for interactions with tissue for further applications, such as biopsy.

### 2.2. Specific Requirements of Biopsy Capsule Endoscope (CE)

The requirements for the practical biopsy endoscopic capsule considered in this study are presented in Table 1. The capsule platform is shaped as a large vitamin pill and is equipped with a camera, light-emitting diodes, wireless communication, and a battery for power supply. The CE should be sufficiently small to be swallowed by humans. Normally, dimensions of approximately 3.0 cm^3^ are suitable for a standard-sized CE [25]. The wireless telemetry module is connected to collect and transfer the image data to the users’ monitors in real time. The camera module must provide clear and high-quality images and have a wide angle of view ranging from 140° to 172° [6]. For extensive utilization at clinical sites, the endoscopic capsule requires multifunctional abilities, such as localization, drug delivery, tattooing, and biopsy. Capsule should be able to perform 5-DOF locomotion with propulsion force at least 50 mN [26,27]. Furthermore, the external electromagnetic system, as a wireless actuator for the CE, must be in the safety range and pose no risk to humans, according to the standard requirements for magnetic-field safety [28]. The biopsy tool should be small enough to be integrated into the capsule body, where the camera, battery, and telemetry module often take up two-thirds of the volume of the capsule body. Additionally, the biopsy tool must be controlled actively by the clinician or operator and be stable during the entire procedure. The mechanism should be able to create a sufficient destructive pressure at the biopsy extraction site (1.2 MPa) [20], and collect a sufficient amount of tissue (1–5 mm^3^) for reliable histological analysis [21,29]. Finally, the biopsy module should consume a small amount of energy (approximately 20% of that consumed by the capsule battery, whose capacity is currently limited to ~475 mWh).

### 2.3. Design of Biopsy Needle CE and Application Scenario

Two common biopsy tools are used clinically: biopsy forceps and biopsy punch needles. In this study, we used the latter for the CE because it is superior to the biopsy forceps. The biopsy needle has played an important role as a medical diagnostic tool. It is inexpensive, simple, highly efficient, and easy to use. The biopsy needle was first used to perform skin biopsies and was then gradually used in intestinal biopsies employing endoscopic instruments to collect samples from neoplasms and intestinal-wall abnormalities [24,30]. The required cutting force of the biopsy punch is smaller than that of the biopsy forceps tool, which was experimentally investigated, as discussed in Section 3.2; therefore, the biopsy punch is more suitable for the CE. According to the clinically verified procedure of the biopsy needle, our idea is to integrate the biopsy punching methodology into a miniaturized CE to achieve minimally invasive diagnosis. This has not yet been investigated, owing to the lack of microfabrication technology and active locomotive ability for CE applications. The proposed technique is developed to acquire biopsy samples in the deep part of the bowels, particularly in the small bowel, where a conventional wire endoscopy cannot access and the commercialized CE is mainly utilized for diagnosis. To realize this idea, several challenges must be overcome. First, the needle must be exposed outside the capsule to contact and collect the targeted tissue. Therefore, the biopsy module should be controllable so that the needle can be extruded once the capsule reaches the target and stowed inside the capsule body during locomotion to prevent damage to organs. Second, the biopsy module should be actuated independently from the locomotion of the capsule robot. Regarding the implementation of internal battery-free biopsy mechanisms, this condition remains as a bottleneck issue for the development of a functional CE. Finally, all advanced CEs may face with the same challenge of a small size fabrication. The biopsy module should be simple and occupy a small portion of the total capsule body.

**Figure 2:**
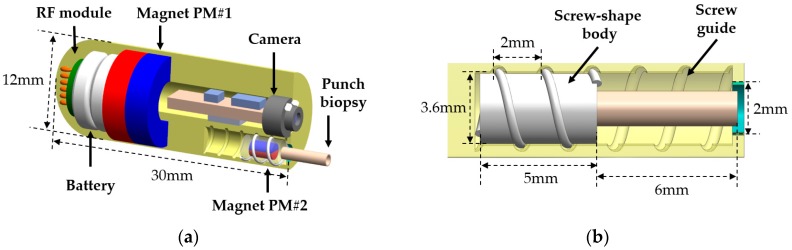
(**a**) Conceptual design of the proposed biopsy CE. (**b**) Design details of the novel punch biopsy module.

Figure 2a depicts the detailed design of the proposed ACE equipped with a punch biopsy module to perform robotic five-DOF motion and micro-linear actuation. The developed capsule has a vitamin-pill shape that is suitable for oral administration. It is composed of a camera module, a battery, a signal communication radiofrequency module, permanent magnets, and a retractable biopsy punch that is used to obtain biopsy tissue. Two permanent magnets—PM#1 and PM#2—are installed in the endoscopic capsule. The former is a ring-type, with an outer diameter, inner diameter, and height of 11, 5, and 6 mm, respectively; it is used for navigation and generation of tissue cutting force. The latter is cylindrical, with a diameter of 2 mm and a length of 3 mm. It is 48 times smaller than the large magnet, to minimize the size of the biopsy module and prevent the magnetic effect of PM#1. This magnet is used for actuating the biopsy punch.

The biopsy module is designed by incorporating a screw mechanism that can extrude and retract the punch needle magnetically. By applying a rotational magnetic field, the extracting or retracting motion of the biopsy tool can be controlled without consuming internal battery power. Figure 2b presents the structure and dimensions of the module. The biopsy needle is connected to PM#2, which is covered by a screw-shaped body. The cover has a pitch of 2 mm, an outer diameter of 3.6 mm, and a length of 5 mm. It can rotate four turns in a clockwise direction to reach its maximum extrude biopsy punch under the rotational magnetic field. At the end of the screw guide, there is a barrier with a diameter of 2 mm, which limits the translation of the magnet and prevents it from falling out. This mechanical stop has no effect on the rotational motion of the magnet; therefore, it does not affect the retrieval of the punch tool. To avoid unexpected extrusion motion of the needle, the rotational magnetic field is disabled during locomotion. The developed mechanism allows the camera to capture the biopsy punch as it extrudes, improving the control of the biopsy punch in the case of unwanted extruding motion.

**Figure 3:**
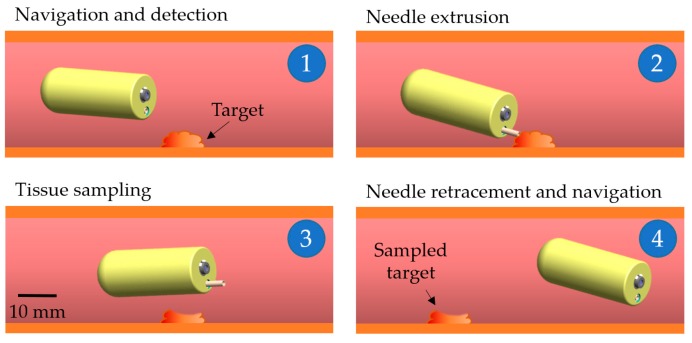
Application scenario of the proposed punch-biopsy CE in the intestine.

Figure 3 presents the application scenario of the proposed biopsy ACE. First, it is operated to scan the digestive tract and reach a targeted lesion. Here, the capsule is aligned in a given direction by a uniform magnetic field. Second, the rotational magnetic field is applied to open the biopsy punch, by exploiting the screw mechanism that converts the rotation motion of PM#2 into translation motion of the biopsy punch. The operator can view the tip of the biopsy punch using the integrated camera. This is one of the advantages of the proposed biopsy CE compared with others, as the entire sampling procedure is visible to doctors. Third, the punch needle is pushed to the target by the external magnetic force, and then magnetic torque is generated to tear the tissue from the target. Finally, ACE retracts the biopsy punch into its body by reversing the direction of the rotational magnetic field used to expose it. Then, the ACE continues the visualization procedure.

### 2.4. Active Locomotion through Electromagnetic Actuation (EMA)

**Figure 4:**
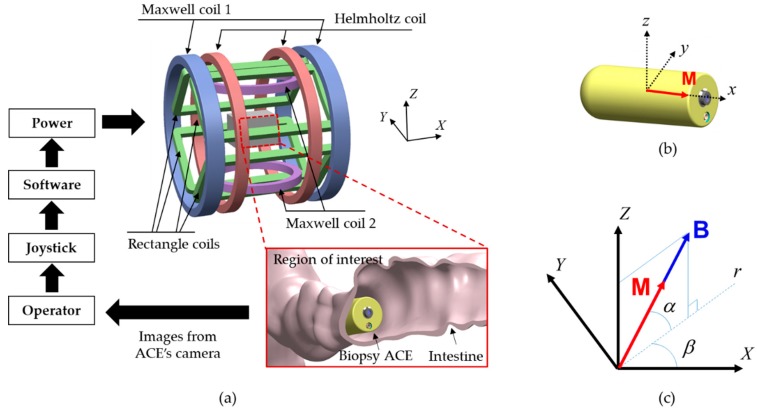
(**a**) Magnetic locomotion system. (**b**) Local coordinate of the CE robot; **M** represents the magnetization vector of the capsule. (**c**) Alignment of the capsule in the world coordinate system; **B** represents the controlled magnetic field, *α* represents the aligned angle between **B** and the *XY* plane, and *β* represents the aligned angle between the *Zr* plane and the *XZ* plane.

The conventional endoscopic capsule is a passive device; thus, its motions are limited to the peristaltic movement of digestive organs. This leads to ineffective diagnosis and therapeutic motion in small tubular organs and unreliability in large digestive organs such as the stomach and colon. Therefore, the motions of the CE must be actively controlled. In this study, we utilized an EMA system to fulfill the requirement of controllable wireless active locomotion for the endoscopic capsule [9]. This EMA system was designed for the manipulation of functional CEs with a maximum magnetic field of 0.09 T and a gradient field of 1.2 T/m. Figure 4a presents the structure of the system, which comprised two pairs of Maxwell coils, a pair of Helmholtz coils, and four rectangular coils. The operators observe the signal transmitted by the CE and control the capsule manually by changing the input parameters using a joystick. The independent control algorithm recently proposed by our group is implemented to compute the electrical currents that must be applied to each electromagnet to generate the desired magnetic field in the ROI [9]. The software manages the power system to supply the required energy to the EMA system.

When the capsule is within the ROI of the EMA system where the magnetic field is controllable, the resulting magnetic torque and force acting on the permanent magnet steer the capsule and push it along the planned path, respectively. Figure 4b depicts the local coordinate system of the CE, with its magnetization vector **M** assigned to the *x*-axis, and Figure 4c depicts the alignment of the magnetic capsule in the world coordinate system. In the controlled plane *Zr*, the capsule is driven by a magnetic torque, as follows:

(1) $$\tau =M\times B=\left[\begin{array}{ccc} i & j & k\\ M_{X} & M_{Y} & M_{Z}\\ B_{X} & B_{Y} & B_{Z} \end{array}\right],$$

where **M** = (*M_X_ M_Y_ M_Z_*)^T^, **B** = (*B_X_ B_Y_ B_Z_*)^T^, and (*i j k*)^T^ is the unit vector of the world coordinate system. Because the magnetic field is proportional to the input current of the electromagnetic coil, the controlled magnetic field **B** can be calculated via a linear combination of 10 coils, as follows:

(2) $$B=\sum_{m=1}^{10}B_{m}=\sum_{m=1}^{10}b_{m}i_{m}=bI,$$

where $b_{m}$ (3 × 1 matrix) and $i_{m}$ represent the magnetic field at the unit current of the *m*^th^ coil and the input current, respectively. **b** (3 × 10 matrix) is the mapping matrix of the magnetic field of 10 coils with respect to the input current vector **I** (10 × 1 matrix). The induced magnetic field aligns the vector **M** along the vector **B**.

The magnetic force pushing the capsule can be calculated using the following equation:

(3) F=(M.∇)B=[∂BX∂X∂BY∂X∂BZ∂X∂BX∂Y∂BY∂Y∂BZ∂Y∂BX∂Z∂BY∂Z∂BZ∂Z]M,

where ∇ is the gradient operator. Using Equations (2) and (3), we can describe the magnetic force in the form of a linear equation with the current vector, as follows:

(4) F=(M.∇)B=[MT∂b∂XMT∂b∂YMT∂b∂Z]I.

Normally, the capsule is pushed along the alignment direction; therefore, the direction of the vector **F** is identical to that of **B**, although the magnitudes differ.

### 2.5. Magnetically Actuated Punching Procedure

**Figure 5:**
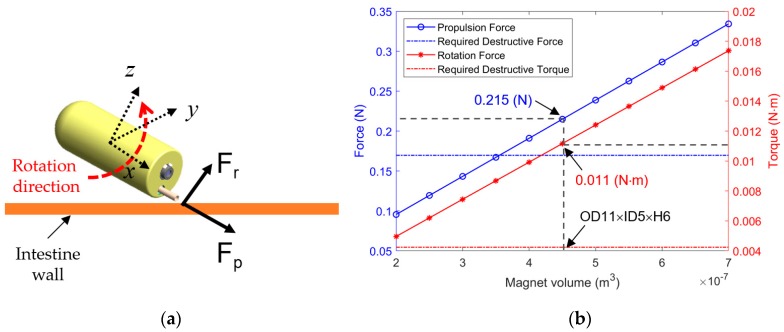
(**a**) Biopsy procedure and cutting force. (**b**) Estimation of the propulsion force and rotation torque based on the simulated volume variation of the PM#1.

The tissue-harvesting process consists of two steps. In the first step, the capsule is pushed against the intestinal wall by a magnetic force F_p_ (see Figure 5a), and the biopsy tool penetrates the tissue. It creates a cutting imprint on the surface of the organ, which can be easily sampled by applying a shear force. In the second step, a magnetic torque created by the rotation motion of the capsule about the *y*-axis (local coordinate system) generates a cutting force F_r_ to tear off the tissue, as shown in Figure 5a.

The punch tool is extruded by screw motion before sampling. The screw motion is created by rotating PM#2 under the rotation magnetic field about the *x*-axis of the capsule coordinate system or the axial axis of the capsule. The rotation of the magnetic field vector **B** with respect to an arbitrary axis **u** by an angle *φ* can be expressed as:

(5) $$R_{u}\left(\varphi \right)B=u\left(u\cdot B\right)+\cos\left(\varphi \right)\left(u\times B\right)\times u+\sin\left(\varphi \right)\left(u\times B\right).$$

In Equation (5), the magnetization vector **M** is collinear to the *x*-axis; thus, the vector **u** can be taken as **M** that is the current posture of the CE. The resultant magnetic torque is exerted on both permanent magnets. Therefore, the amplitude of the rotation magnetic field should be small enough to reduce the vibration of the capsule body but large enough to actuate PM#2.

Once the punch needle is fully exposed, the operator can observe the target position and the needle through the camera of the capsule. The capsule is steered to direct the biopsy tool toward the target. Then, the magnetic force in Equation (4) is generated for the tool to penetrate the tissue, creating a cut imprint on the mucosal layer. The shear stress induced by the biopsy tool must be larger than the standard destructive stress of the mucosa layer, which is given as $\tau_{des}$ = 1.2 MPa [20]. Therefore, the required cutting force at the tip of the biopsy punch for collecting the tissue can be obtained as follows:

(6) $$\tau_{ex}=\frac{F_{cut}}{Area}=\frac{F_{cut}}{s\times \pi \times d_{punch}}\geq \tau_{des},$$

(7) $$F_{cut}=\tau_{des}\times s\times \pi \times d_{punch},$$

where $\tau_{ex}$ represents the shear stress exerted on the tissue by the biopsy punch, $F_{cut}$ represents the cutting force acting on the tissue, *s* represents the sharpness, and *d_punch_* represents the diameter of the tip of the biopsy punch. In this study, we used a punch tool with outer and inner diameters of 1.5 and 1.2 mm, respectively, and the sharpness of the tool tip was 0.03 mm. Therefore, the required cutting force $F_{cut}$ was estimated to be at least 0.17 N (*d_punch_* = 1.5 mm).

Finally, the rotation motion of the capsule body is used to tear off the target. The required magnetic torque can be estimated as follows:

(8) $$\left\|\tau \right\|=F_{cut}\times d,$$

where *d* = 25 mm is the distance from the center of PM#1 to the tip of the fully extruded punch tool. Accordingly, the capsule must be able to create a minimum of 0.0042 N∙m of physical torque.

The propulsion force and rotational torque of the capsule body were estimated for various volumes of the permanent magnet to optimize the dimensions of the permanent magnet PM#1. Figure 5b presents the results of the simulation, with a magnetization of the Neodymium permanent magnet with a magnetization value of *M* = 955,000 A/m. In this simulation, we used a gradient magnetic field of 0.5 T/m and a magnetic field of 0.03 T for the propulsion force and torque, respectively. The inner diameter of PM#1 was fixed to 5 mm to leave space for the camera and its signal wire. We simulated the magnetic response with various values of the outer diameter and height. We selected a magnet with an outer diameter of 11 mm and a height of 6 mm, which is denoted as PM#1 in Figure 2. This magnet can be placed inside the capsule body and generates 0.215 N of propulsion force and 0.011 N∙m of torque, which are both higher than the values required for cutting. Additionally, the destructive stress of the intestinal wall is three to four times larger than that of the submucosa layer [31]. This choice is adequately safe to protect the intestinal wall from perforation.

## 3. Experiment and Results

**Figure 6:**
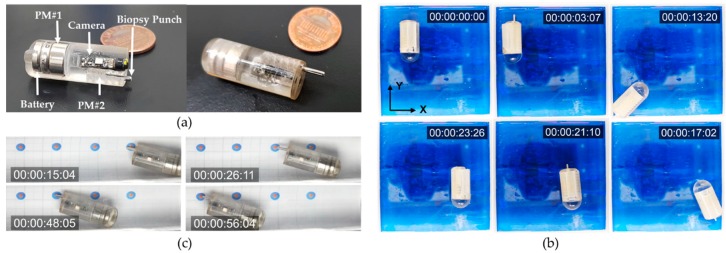
(**a**) Components of the active capsule endoscope (ACE) and biopsy module. (**b**) Needle control and locomotion experiments in a large space with small friction in chronological sequence. (**c**) Targeting experiment for the given markers on the phantom. A real-time watch was added, which shows the time in the format hour:min:sec:msec.

The designed ACE with the biopsy punch mechanism was fabricated and assembled for experimental evaluation, as shown in Figure 6a. The ACE chassis was fabricated using a rapid prototyping three-dimensional (3D) printer (Object 30 Pro, Stratasys Direct Manufacturing Ltd., Los Angeles, CA, USA) with VeroClear RGD810 (Stratasys Direct Manufacturing Ltd.) as the resin. The disposable biopsy punch was purchased from Kai Medical (Kai Industries Co., Ltd., Seki, Japan). It had outer and inner diameters of 1.5 and 1.2 mm, respectively, and the sharpness of the tool tip was 0.03 mm. There were two permanent magnets inside the body of the capsule. The large permanent magnet, which was used for locomotion and tissue extraction, was ring-shaped, with a height, outer diameter, and inner diameter of 6, 11, and 5 mm, respectively. The small permanent magnet connected to the biopsy punch had a cylindrical shape and was 2 mm in diameter and 3 mm in length. The magnets were separated by a distance of 5 mm, which was a safe distance for a small interaction force between them and for them to be independently controlled. The assembled biopsy capsule had a diameter and length of 12 and 30 mm, respectively.

The experimental setup is illustrated in Figure 4a. The operator controlled the capsule manually by adjusting the steering angles, amplitude of the magnetic field, and applied force using a joystick (Extreme 3D Pro, Logitech, Lausanne, Switzerland). A human–machine interface (HMI) was built in LabVIEW 2017 (National Instruments, Austin, TX, USA) with a developed control algorithm. A power system (including MX15 (5 units) and 3001LX (5 units) from California Instruments, San Diego, CA, USA) that can generate currents of up to 20 A was used to power the EMA system. The power system was connected to a central computer via a general-purpose interface bus and was controlled by the HMI. All the input parameters given by the joystick and keyboard were updated in the HMI in real time. The biopsy CE prototype and tested objects were placed in the ROI of the EMA system. Two cameras (C920, Logitech) were used to capture images from top and side views.

### 3.1. Biopsy Needle Control and Locomotion of ACE

A set of in-vitro tests was performed to evaluate the efficiency of the control of the magnetic field for biopsy needle actuation and capsule locomotion. Because the screw-shaped body and screw guide were printed by the 3D printer and the friction coefficient was difficult to measure, we experimentally determined the appropriate amplitude of the rotation magnetic field to extrude the needle. It should be sufficient to actuate the screw mechanism and small enough to prevent the vibration of the capsule body. By adjusting the magnetic-field amplitude with step inputs of 0.2 mT, a range of 4–6 mT (weaker than the alignment magnetic field of 15 mT) was used to activate the biopsy needle.

First, the ACE was placed in an acrylic phantom in the ROI of the EMA system. Experiments were performed in air, and the capsule was manipulated manually by the user. Figure 6b shows the needle manipulation process and locomotion in chronological sequence. Initially, the users aligned the capsule along the desired direction (*Y-*axis in this test) using a magnetic field of 15 mT and then extruded the punch biopsy using the rotation magnetic field with an amplitude of 4 mT. After 3 s, the ACE prototype could extrude the biopsy punch from its body without affecting the posture of the capsule. However, the position of capsule was changed slightly, owing to the tubular shape of the capsule body, which caused undesired rolling motions. Subsequently, the capsule was moved to arbitrary positions along the *Y*-axis and *X*-axis; the rotation magnetic field was disabled in this phase to avoid unwanted motion of the needle. The biopsy punch was stable during the locomotion. Finally, the rotation magnetic field was applied in the reverse direction to retract the biopsy tool into the body of the capsule. By controlling the external magnetic field, we could actuate the needle and move the capsule independently. It took approximately 3 s to extrude and retract the needle.

Second, a test was performed to evaluate the targeting accuracy of the biopsy tool under the open-loop control system. We conducted targeting experiments using a phantom model with a known geometry and targeting markers. The phantom had a cylindrical shape with a millimeter grid. Four double-rounded targets were marked inside the phantom: 1-mm-radius red inner targets and 2.5-mm-radius blue outer targets. The ACE with the pre-extruded needle was placed in the phantom and controlled to approach all the red targets. The capsule was first aligned in the desired direction by the magnetic field and was then pushed forward/backward by the magnetic force. The user could manually increase or decrease the magnetic force to obtain large or small movement steps of the capsule, respectively. As shown in Figure 6c, we successfully drove the biopsy punch to reach all four destinations. The biopsy tip contacted the targets with high accuracy, within a range of 2.5 mm from the target point. To increase the precision, a closed-loop control strategy can be applied, where a tracking system is integrated with the EMA system to determine the location and orientation of the ACE.

### 3.2. Cutting Force of Punching Needle Biopsy

To verify the cutting force of the proposed needle punching biopsy analyzed in Section 2.5, we measured the propulsion force and rotational force of the biopsy capsule generated by the EMA system. The rotation force is created by the magnetic field, and the propulsion force is generated by the gradient field, which is also used to move the biopsy CE.

**Figure 7:**
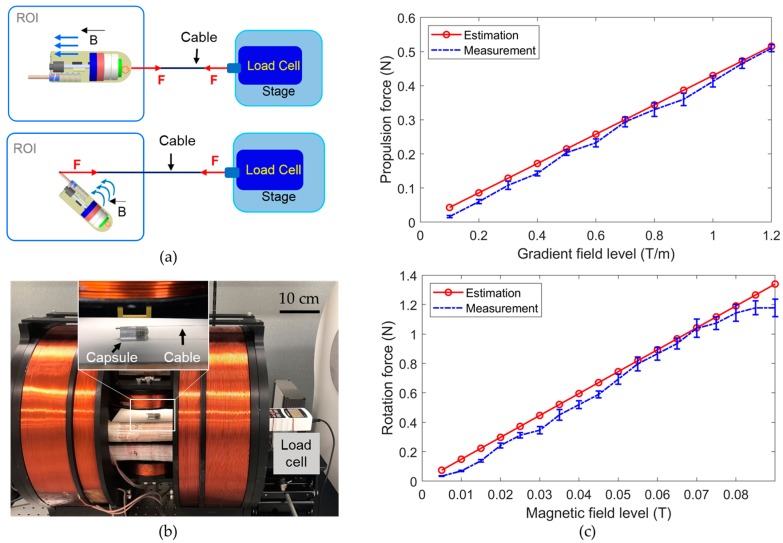
(**a**) Experimental setups for measuring the propulsion force (top) and rotation force (bottom). (**b**) Experimental setup. (**c**) Propulsion force and rotation force of the ACE.

Figure 7a,b present the configurations and experimental setup for measuring the propulsion and rotational forces. A linear stage with a load cell (Advanced Digital Force Gauges Series 5, Mark-10, Copiague, NY, USA) was set up and connected to the ACE through a cable. The cable was connected to the capsule body for measurement of the propulsion force and to the biopsy tool tip for measurement of the rotational force. The ACE was placed in the ROI of the EMA system. The driving and steering forces were measured at various levels of the gradient field and magnetic field, respectively. Figure 7c shows the measured forces and the estimation results obtained using Equations (2) and (4). The values measured in the experiments were close to the values estimated in the simulation. Therefore, the calculation in Section 2.5 to optimize the size of PM#1 is reliable. The minor differences between the measured force and the simulated force could be caused by the frictional force between the ACE and the ROI surface and systematic errors. The propulsion force was measured to be approximately 0.2 N at a gradient field of 0.5 T/m, and the rotation force was approximately 0.34 N at a magnetic field of 0.03 T. Both were larger than the required cutting force of 0.17 N, indicating the feasibility of biopsy tissue extraction using magnetic actuation. Moreover, the operator can use stronger gradient and magnetic fields to generate stronger cutting forces, according to the magnetic force and torque curves in Figure 7b, if necessary.

**Figure 8:**
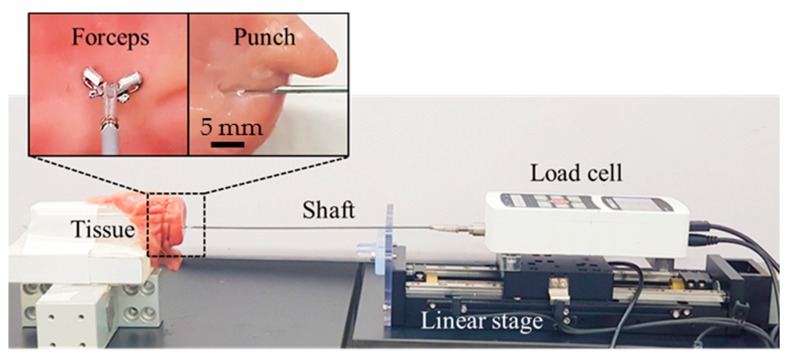
Experimental setup with commercial biopsy forceps and the biopsy punch.

Additionally, in a comparative study, we measured the cutting forces of the biopsy punch and the biopsy forceps commonly used at clinical sites by endoscopy clinicians, to show that it is more beneficial to integrate punch needle into CE for tissue extraction. We measured the cutting force of the clinically used endoscopic biopsy forceps (Olympus, Tokyo, Japan, diameter of 1.8 mm) and the biopsy punch, as shown in Figure 8. The measured forces from the biopsy forceps and the biopsy punch were in the ranges of 1.0–1.2 N and 0.3–0.4 N, respectively. The measured force of the biopsy punch was smaller than that of the biopsy forceps, because of several factors, such as the sharpness of the tool tip, the size of the extracted tissue, and the clamping force of the forceps wire. Regardless of the actuation method, punch needle is more suitable for ACE than biopsy forceps in terms of extraction force. Accordingly, it is confirmed that the developed biopsy punch mechanism with external EMA can generate enough force to obtain tissue samples from the intestine.

### 3.3. Ex-Vivo Experiments

**Figure 9:**
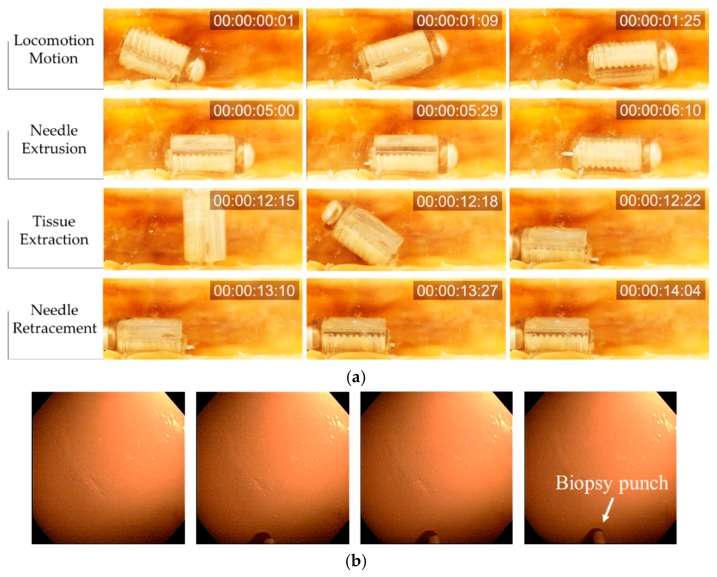
(**a**) Ex-vivo locomotion and biopsy experiment in chronological sequence. A real-time watch was added, which shows the time in the format hour:min:sec:frame. (**b**) Images obtained using the camera of the CE as the biopsy punch was extruded.

The performance of the developed biopsy CE was evaluated through ex-vivo experiments. We used a piece of porcine small intestine (purchased from local market Malbau, Gwangju, South Korea) and fixed it in a semi-cylindrical tube. The small intestine and the biopsy ACE device were placed at the center of the ROI of the EMA system. Figure 9a presents the complete biopsy procedure in chronological sequence, with the target at the middle of the intestine segment.

First, the ACE was controlled to move to the target point. An external electromagnetic field was generated to align the ACE in the desired direction, and then a gradient field was used to push it in the desired direction. The rotation magnetic field was disabled during the locomotion process to prevent undesired motion of the biopsy needle. Once the ACE reached the destination, the gradient field was turned off, and the rotational magnetic field was generated to extrude the needle. As mentioned previously, one of the advantages of the proposed biopsy CE over other tools is that the extrusion step and the entire sampling procedure can be viewed using the camera of the capsule. Figure 9b shows the status of the punch needle during extrusion. The image was obtained using the camera of the capsule. Next, the rotation magnetic field was disabled to prevent undesired rotation motion of the biopsy punch. The ACE was then aligned and pushed against the intestinal wall to create an imprint. The magnetic torque was used to extract tissue, as described previously. Finally, the needle was retracted into the body of the capsule by reversing the direction of the rotation magnetic field used to open it.

**Figure 10:**
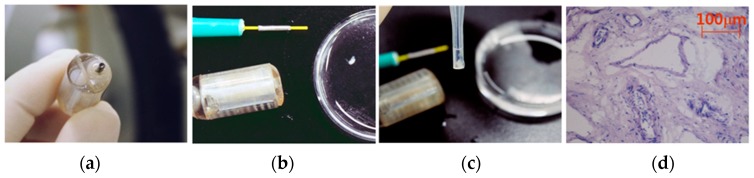
Biopsy sample examination processes. (**a**) ACE with the biopsy tool after the ex-vivo experiment. (**b**) Removal of the biopsy punch from the capsule. (**c**) Collected tissue fixation before analysis. (**d**) Biopsy tissue under a microscope.

The proposed biopsy CE could be controlled to move to target positions, and the developed biopsy mechanism was successful in sampling biopsy tissue. There was no perforation on the intestine. To validate the quality of the extracted samples, we conducted an examination, as shown in Figure 10. The disposable biopsy punch was removed, and the samples were collected for histological analysis. The average size of the collected samples was 1 mm × 1.3 mm × 1.5 mm, which was sufficient for the histological analysis in Table 1.

For the investigation of the harvested biopsy tissues, hematoxylin and eosin (H&E) staining was performed [32]. After the tissues were harvested, they were stored in phosphate-buffered saline (PBS) and then embedded in an optimal-cutting temperature compound, followed by storage at −80 °C until use. Next, tissue sections approximately 10 μm thick were mounted onto slides fixed with pre-cooled 4% paraformaldehyde for 5 min and washed with PBS for 10 min. Subsequently, the sections were stained with H&E, via the standard protocol, and examined using optical microscopy [33]. As shown in Figure 10d, the nuclei were stained deep blue, and the cytoplasm was stained light red.

## 4. Discussion

In this study, as a critical application for clinicians, a biopsy modality based on an actively controlled CE using biopsy needle punching was developed. This was the first trial to transfer a clinical biopsy tool into a miniaturized CE, which allows the collection of biopsy tissue in deep regions of the small intestine. The developed biopsy CE has several advantages. The biopsy process is manipulated magnetically by an external control system; therefore, it does not consume the energy from the internal battery of the capsule, which has limited power. The new design allows the operators to view the entire biopsy procedure, including the needle actuation and biopsy punching motions. This feature is a significant advantage of our design, as it can enhance the retrieval rate of biopsy tissue. Therefore, the proposed tool has the potential to expedite the detection of abnormal tissues and improve the diagnosis accuracy.

The proposed biopsy module is simple and easy to operate. By utilizing a small magnet covered by a screw-shaped body, the biopsy module can be actuated independently from the locomotion. The number of components of the biopsy module is small, making it small and inexpensive to fabricate. Additionally, the screw mechanism can be used for other endoscopic capsule applications in which the tools must be kept inside the capsule body during locomotion for safety and exposed at desired location (e.g., sensing, drug injection, and tattooing).

Although the proposed mechanism can facilitate endoscopy and biopsy procedures, it has limitations for application at clinical sites. Performing multiple biopsies in different target regions is impossible because of the space limitation. However, the proposed mechanism can be used to biopsy multiple tissues at the same target or along a segment of the intestine under suspicious or abnormal conditions for special cases, such as aged patients who may have difficulties with conventional endoscopy due to the discomfort and side effects. The proposed capsule can function easily in tubular organs (small and large intestines). It might be difficult to operate in the stomach owing to the folding structures and high-curvature parts (e.g., fundus, antrum, and top of stomach). In this study, we did not quantify or model the effect of the intestinal environment for the precise control of the capsule motion and posture inside the intestine. Additionally, the motions of the living body and the pressure of internal organs were not considered in the ex-vivo tests.

In the future, the developed biopsy capsule will be tested in animals, followed by clinical trials. The working environments, which affect the control performance, will be analyzed for in-vivo tests. The in-vivo experiments will be conducted on subjects to validate the proposed system with the scaled up system. A feedback system and advanced control techniques will be integrated with the EMA system to track the capsule and improve the control performance of the system in the living body, where there are noise and uncertain factors (e.g., respiration motion, pressure from internal organs) [34,35]. Advanced applications of the linear motion mechanism driven by an external EMA system will be researched in accordance with clinical demands.

## 5. Conclusions

In this paper we have presented a novel retractable needle punching biopsy methodology integrated with an ACE that could perform robotic movement for the histological analysis of intestinal diseases. Numerical analysis and in-vitro tests proved the feasibility of utilizing an available medical biopsy punch for the ACE to sample tissues in the intestine. The developed screw mechanism was actuated magnetically to extrude and retract the biopsy tool without affecting the posture of the capsule. In ex-vivo tests, we successfully demonstrated a full intestinal diagnosis procedure, including locomotion for visual inspection, needle actuation, and tissue acquisition.

Regarding clinical applications, the proposed biopsy CE with the noninvasive monitoring ability and microscale-functionality could operate in the intestinal tract and provide a view of organs for drug delivery or tissue sampling. The two main contributions of this work are summarized as follows: (1) we demonstrated a novel battery-free screw mechanism for an ACE driven by an external EMA system, and (2) we performed the first trial integrating a medical biopsy punch with an ACE to obtain intestinal samples. In conclusion, we demonstrated the potential applicability of the biopsy punch with the developed screw mechanism to the recently developed five-DOF locomotive active capsule for gastrointestinal-tract diagnosis.

## Figures and Tables

**Table 1:** Design specifications of a biopsy capsule endoscope (CE).

Design Specification	Target Value	References
Capsule Size	3.0 cm^3^	[25]
Images	320 × 320 pixels	[6]
Locomotion	5 DOF	[26]
Propulsion Force	50 mN	[27]
Biopsy Tissue Volume	1–5 mm^3^	[21,29]
